# The Population Dynamical Implications of Male-Biased Parasitism in Different Mating Systems

**DOI:** 10.1371/journal.pone.0000624

**Published:** 2007-07-18

**Authors:** Martin R. Miller, Andrew White, Kenneth Wilson, Michael Boots

**Affiliations:** 1 Department of Animal and Plant Sciences, University of Sheffield, Sheffield, United Kingdom; 2 Department of Mathematics and the Maxwell Institute for Mathematical Sciences, Heriot-Watt University, Edinburgh, Scotland; 3 Department of Biological Sciences, Lancaster Environment Centre, Lancaster University, Lancaster, United Kingdom; Yale University, United States of America

## Abstract

Although there is growing evidence that males tend to suffer higher levels of parasitism than females, the implications of this for the population dynamics of the host population are not yet understood. Here we build on an established ‘two-sex’ model and investigate how increased susceptibility to infection in males affects the dynamics, under different mating systems. We investigate the effect of pathogenic disease at different case mortalities, under both monogamous and polygynous mating systems. If the case mortality is low, then male-biased parasitism appears similar to unbiased parasitism in terms of its effect on the population dynamics. At higher case mortalities, we identified significant differences between male-biased and unbiased parasitism. A host population may therefore be differentially affected by male-biased and unbiased parasitism. The dynamical outcome is likely to depend on a complex interaction between the host's mating system and demography, and the parasite virulence.

## INTRODUCTION

There is now considerable evidence that the two sexes differ in their rates of parasitism. Male-biased parasitism is thought to be much more common [1]–[4], although higher levels of infection with blood parasites have been observed in female birds [5]. Males may be more susceptible to infection and/or they may transmit infection more easily than females. It has been pointed out, however, that in populations where males have higher levels of infection, they are also likely to be responsible for most of the transmission to females [6]. There are several reasons why males may be more prone to parasitism. Sexual size dimorphism in mammals is often strongly correlated with higher male mortality; a comparative study identified positive correlations between male-biased parasitism and the degree of sexual selection, and between male-biased parasitism and male-biased mortality [7]. At least two explanations have been suggested to explain these patterns. One is that the larger body size and/or higher growth rates of males may make them easier targets for parasites [7]. Another is that males are more susceptible to parasitism due to the immunodepressive effects of the androgenic hormones (e.g. testosterone in vertebrates) required for increased growth and reproductive effort [7], [8]. Indeed, since males tend to gain fitness largely through reproductive competition and females through enhanced longevity, it is predicted that males might invest less in costly immune mechanisms in order to divert resources into traits that enhance competitive ability [9], [10].

Given that male-biased parasitism appears to be ubiquitous, it may have widespread implications for population dynamics. Ecological models have the potential to exhibit extremely complicated behaviour [11]–[13] although natural populations tend to be relatively stable [14], [15]. Here, we investigate the effects of male-biased parasitism on the population dynamics. A common approach in theoretical studies is to assume that the population dynamics can be understood by examining only one of the sexes in isolation. This approach can be justified if males and females have similar life cycles, or if one sex is completely dominant, in which case the dynamics are independent of the abundance of the other [16]. However, there are often significant demographic differences between the sexes [17]–[21]. For example, mammalian species with polygynous mating often show strong sexual selection for larger males, which tend to have higher mortality [7], [22]–[24]. The assumption of complete dominance also fails in many cases, where an uneven sex ratio may constrain reproduction due to limited availability of the scarcer sex. A consideration of sexual reproduction and the differences between sexual classes may have a profound effect on the dynamics, and appropriate ‘two-sex’ models should therefore consider males and females separately [16], [25].

Modelling separate classes for each sex has important implications in terms of the dynamics, because births are dependent on both sexes. Caswell and Weeks [16] analysed an explicit two-sex model where births were determined by a ‘harmonic mean’ function, according to which reproduction depended on the ratio of males to females, and declined to zero in the absence of either sex. This birth function could be modified to accommodate polygynous or polyandrous mating systems. The authors also showed how demographic differences between the sexes could lead to a range of complex behaviour, from periodic or quasi-periodic cycles, to apparently chaotic dynamics [16].

Following on from this, Lindstrom and Kokko [26] compared the relative stability of sexual and asexual populations, in a model that included both polygyny and demographic sex differences. As the intrinsic growth rate increased, the asexual population exhibited a period-doubling route to chaos. With no demographic differences between the sexes, a monogamous population exhibited greater stability than an asexual one, and polygynous populations were stable to a similar degree as asexual ones. Where males experienced higher crowding, this had a destabilizing effect: chaos was observed at higher growth rates, and the dynamics no longer showed period-doubling bifurcations. Polygynous populations with higher male crowding were highly unstable, exhibiting chaos or cycles at all but very low growth rates.

Here we build on an established theoretical framework and examine how male-biased parasitism affects dynamical stability, compared to an unbiased parasite, under different characteristic mating structures. In line with previous two-sex models, we include the effect of demographic sex differences. The basic model is derived from the model of Lindstrom and Kokko [26] for a disease-free sexual population, and the host-parasite framework of May and Anderson [27]. Our epidemiological model therefore incorporates the specific demography of the individual male and female sub-populations. In particular, we examine the effect of male-biased parasitism at different levels of case mortality.

## MODEL

The model of May and Anderson [27] describes a host-microparasite interaction with discrete, non-overlapping host generations. Disease epidemics occur within a cohort such that only surviving hosts are able to reproduce. Here, we generalize the model to include both male and female hosts. For each sex, the densities of uninfected, infected and immune hosts are denoted by *S_i_*, *I_i_* and *R_i_* respectively (here density is defined as the number of individuals per unit area, where the area is assumed to be constant). The total density of each sex is therefore *N_i_* = *S_i_*+*I_i_+R_i_*. The epidemiological dynamics are described by the following equations:
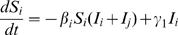
(1)

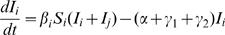
(2)

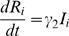
(3), where *m* denotes males and *f* denotes females (*i*≠*j*). The transmission rate of infection is β*_i_* (this allows for different susceptibilities to infection for males and females, but both types of host are equally infectious). There is an increased death rate due to the disease (virulence) given by α. Infected hosts recover to the susceptible class (at rate γ_1_) or to the immune class (at rate γ_2_). These symbols are summarised in Table 1.

**Table 1:** Definition of symbols.

Symbol	Definition
*i*	Sex
*S_i_*	Density of uninfected hosts
*I_i_*	Density of infected hosts
*R_i_*	Density of immune hosts
*N_i_*	Total host density
*S_i_*_,*∞*_	Density of uninfected hosts (end of cohort)
*I_i_*_,*∞*_	Density of infected hosts (end of cohort)
*R_i_*_,*∞*_	Density of immune hosts (end of cohort)
*N_i_*_,*∞*_	Total host density (end of cohort)
ρ*_i_*	Disease prevalence
*B*	Number of births
β*_i_*	Transmission rate
α	Virulence
γ_1_	Recovery rate to susceptible
γ_2_	Recovery rate to immune
δ	Rate of vertical transmission
*v*	Proportion of infected offspring
χ	Case mortality
μ*_i_*	Vulnerability to crowding
*h*	Harem size
*k*	Fecundity

The condition for the host population to support the pathogen is that its basic reproductive ratio (*R*_0_) exceeds unity [28], [29]. Since we are considering a heterogeneous population, the reproductive ratio will depend on the parasite fitness in both males and females and on their individual densities [30]:
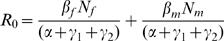
(4)Throughout the analysis, it is assumed that *R*_0_>1. The disease will therefore persist and cause an epidemic. At the end of a given cohort, the epidemic is assumed to have completely run its course, such that there are no infected individuals left in the population. Defining (1−ρ*_i_*) as the proportion of hosts who remain susceptible after the epidemic [27], the densities at the end of the cohort are:
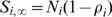
(5)

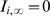
(6)

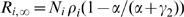
(7)The total density of surviving hosts is therefore:
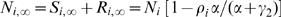
(8)Reproduction occurs according to the harmonic mean function [16], [25]. The number of births, *B*, therefore depends on the densities of both males and females at the end of the previous cohort, and births will fall to zero in the absence of either sex:
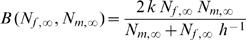
(9)


The parameter *h* gives the average harem size. With monogamous mating, *h = *1, and males and females are equally important in terms of births. Values of *h* greater than one correspond to polygynous mating, where the birth rate is more dependent on females [16], [26]. The population is also assumed to experience density-dependence of the Moran-Ricker type [31], [32], as employed by Lindstrom and Kokko [26]. Population growth is therefore limited at high densities by intra-specific crowding, which for simplicity manifests in terms of increased infant mortality. Assuming births are equally likely to be of either sex, the population densities in the next cohort are:
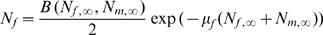
(10)

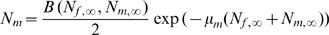
(11)Note that males and females may experience different levels of crowding, as measured by the parameters μ*_f_* and μ*_m_* respectively.

The model suggested by May [33] implicitly assumes that infection persists from one cohort to the next via repeated inoculation from some external source. The model of Koella and Doebeli [34] instead assumes that a small proportion of hosts are infected at the beginning of each cohort due to vertical transmission to offspring. We follow this approach and assume that the proportion of infected offspring, *v,* depends on the proportion of surviving females who recover from infection, and also on the parameter δ measuring the efficacy of vertical transmission:
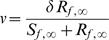
(12)This assumption is made to link the level of infection in one cohort to the level in the previous cohort, in order to allow the parasite dynamics to develop over several generations. It reflects transmission across the cohorts through any method including environmental contamination and infected individuals surviving to infect in the following cohort, as well as any direct vertical transmission. The equilibrium densities (5)-(7) were determined using computer simulations of the differential equations (1)-(3) until the system reached a stable state. Reproduction then occurred according to equations (10)-(11), with a proportion, 1−*v*, offspring classified as susceptible and a proportion, *v*, classified as infected (according to equation 12). This process was repeated 120 times to eliminate the initial transient effects and the population densities were then plotted for the last 20 iterations. From the coincidence of the consecutive values it can be seen whether the system converges to a stable point, limit cycle, or displays other complex behaviour. Note that our model clearly delineates the periods of reproduction and mortality. Juveniles do not experience any mortality due to the disease, which only manifests as virulence once an individual has reached maturity. There is no sterility or reduced fecundity due to vertical transmission.

## RESULTS

We begin by reproducing the results of Lindstrom and Kokko [26], showing how sexual reproduction itself affects dynamical stability. Unbiased parasitism is then added to the basic model, characterized by equal transmission rates of infection to each sex. Finally, the effect of male-biased parasitism is investigated, represented as a higher transmission rate of infection to males (and a correspondingly lower transmission to females, in order to maintain a constant infection rate overall). We investigate the impact of parasitism at different levels of virulence, expressed in terms of the case mortality due to infection, χ = α/(α+γ_1_+γ_2_).

### Disease-Free Host Population

**Figure 1:**
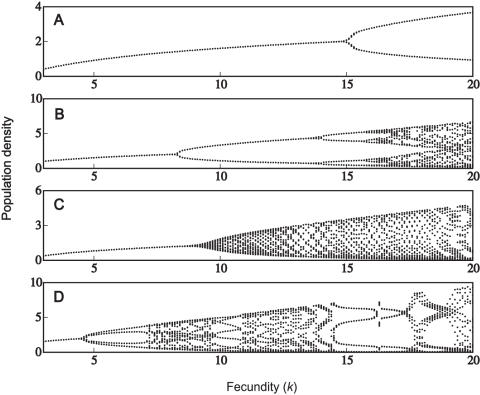
Population dynamics in the absence of parasitism. (A) *h* = 1, μ*_f_* = μ*_m_* = 1; (B) *h* = 10, μ*_f_* = μ*_m_* = 1; (C) *h* = 1, μ*_f_* = 0.4, μ_m_ = 1.6; (D) *h* = 10, μ*_f_* = 0.4, μ*_m_* = 1.6. The rate of vertical transmission is δ = 0.2.

To investigate the effects of parasitism (male-biased or otherwise) the dynamical behaviour in the absence of disease needs first to be established. This has been discussed in depth by Lindstrom and Kokko [26], and the main results are reproduced in Figure 1. A monogamous sexual population with no density-dependent differences between the sexes exhibits the highest degree of stability (Fig. 1A). The inclusion of polygynous mating destabilizes the dynamics; however, the simple period-doubling route to chaos is preserved (Fig. 1B). Returning to the monogamous mating system, but now assuming that males are more vulnerable to crowding, the dynamics become highly unstable at higher fecundities and no longer follow the period-doubling route to chaos (Fig. 1C). A polygynous population that experiences a higher level of male crowding exhibits complex dynamics for most levels of fecundity, with alternating regions of chaos and limit cycles (Fig. 1D).

### Unbiased Parasitism

**Figure 2:**
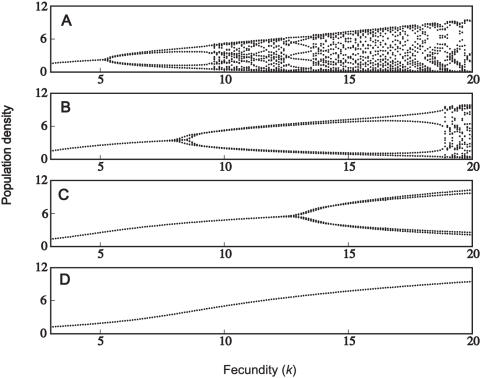
Population dynamics with unbiased parasitism. (A) α  = 0.08, γ*_1_* = γ*_2_* = 0.46; (B) α  = 0.3, γ*_1_* = γ_2_ = 0.35; (C) α  = 0.5, γ*_1_* = γ*_2_* = 0.25; (D) α = 0.7, γ*_1_* = γ*_2_* = 0.15. Other parameters are: β*_f_* = β*_m_* = 1.2, *h* = 10, μ*_f_* = 0.4, μ*_m_* = 1.6 and δ = 0.2.

We now examine the effect of adding parasitism to each of the model systems. Initially, we assume a low case mortality such that χ = 0.08 (infected individuals have a 92% chance of recovery). There is very little effect on the dynamics at this low mortality rate. This is shown in Fig. 2A, illustrating the dynamics for a polygynous mating system with demographic differences (*c.f*. Fig. 1D). However, higher case mortalities (χ) generally increase the stability of the system, as the initial bifurcation shifts further towards the right (Figs. 2B–2D). For high enough mortality rates, the populations are completely stabilized (over the given range of fecundities). Although this behaviour is illustrated for a polygynous mating system with demographic differences, the results are qualitatively the same for all four mating structures: increasing case mortality stabilizes the dynamics for higher values of the bifurcation parameter (fecundity).

The pattern of increasing stability with higher case mortality was found to hold true only up to a point. Very high case mortalities (generally in excess of 95%) tended to promote cyclic dynamics. For case mortalities in excess of 99%, the system exhibited high-period cycles or chaos. This agrees with the model of May [33], which predicted chaotic dynamics for an asexual population. At such high case mortalities, the dynamics appear to be determined almost entirely by the epidemiology, such that there is very little effect of mating system and/or host demography.

### Male-Biased Parasitism

**Figure 3:**
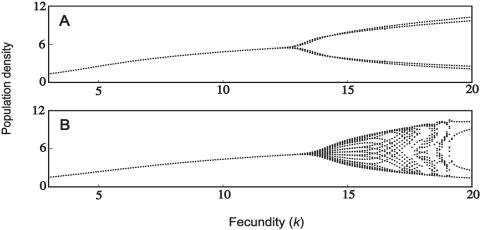
Population dynamics with male-biased parasitism. (A) β*_f_* = β*_m_* = 1.2; (B) β*_f_* = 0.6, β*_m_* = 1.8. Other parameters are: *h* = 10, μ*_f_* = 0.4, μ*_m_* = 1.6, α = 0.5, γ*_1_* = γ*_2_* = 0.25 and δ  = 0.2.

Next we investigate male-biased parasitism, manifested as a higher transmission rate to males (β*_m_*), and a correspondingly lower transmission rate to females (β*_f_*). The overall ‘force of transmission’ (β*_m_*+β*_f_*) is held constant to allow comparison with the unbiased case (β*_m_* = β*_f_*). At low case mortality rates, there appears to be very little difference between male-biased and unbiased parasitism - in both cases the population dynamics are unaffected by the addition of disease (*c.f.* Fig. 2A). At higher case mortalities, however, we identified significant differences between male-biased and unbiased parasitism. An example is shown in Figure 3. At high fecundity, unbiased parasitism results in two- or four-period cycles (Fig. 3A); the periodicity is much higher with male-biased parasitism (Fig. 3B). It is also worth noting that these cycles are of much higher period than is observed in the absence of parasitism (Fig. 1D; 14.5<*k*<17.5). Male-biased parasitism therefore has the potential to destabilize its host population.

**Figure 4 pone-0000624-g004:**
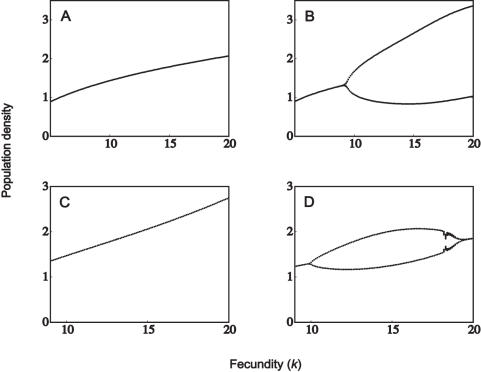
Population dynamics with unbiased and male-biased parasitism. (A) *h* = 1, μ*_f_* = μ*_m_* = 1, α = 0.95, γ*_1_* = γ*_2_* = 0.025, β*_f_* = β*_m_* = 1.2; (B) same as (A) except β*_f_* = 0.4 and β*_m_* = 2; (C) *h* = 1, μ*_f_* = 0.4, μ*_m_* = 1.6, α = 0.8, γ*_1_* = γ*_2_* = 0.1, β*_f_* = β*_m_* = 1.2; (D) same as (C) except β*_f_* = 0.6 and β*_m_* = 1.8. The rate of vertical transmission is δ = 0.2.

**Figure 5 pone-0000624-g005:**
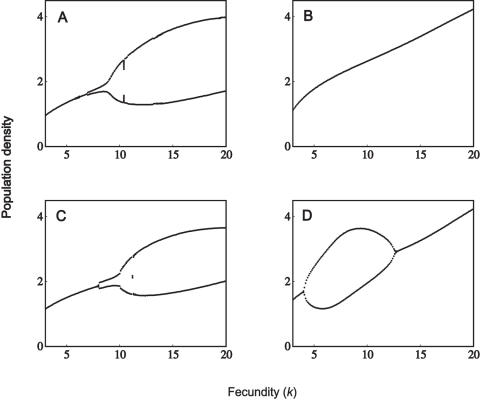
Population dynamics with unbiased and male-biased parasitism. (A) *h* = 10, μ*_f_* = μ*_m_* = 1, α = 0.97, γ*_1_* = γ*_2_* = 0.015, β*_f_* = β*_m_* = 1.2; (B) same as (A) except β*_f_* = 0.6 and β*_m_* = 1.8; (C) *h* = 10, μ*_f_* = 0.4, μ*_m_* = 1.6, α = 0.97, γ*_1_* = γ*_2_* = 0.015, β*_f_* = β*_m_* = 1.2; (D) same as (C) except that β*_f_* = 0.6 and β*_m_* = 1.8. The rate of vertical transmission is δ = 0.2.

Whether male-biased parasitism results in different dynamics may also be dependent on the mating system of the population. This is shown in the following examples (the parameter combinations have been specifically chosen to illustrate the differences). Firstly, monogamous populations may be relatively less stable compared to an unbiased parasite (Fig. 4); these dynamics can also be compared with the uninfected population (Figs. 1A, 1C). We also identified differences under polygynous mating systems: male-biased parasitism may result in greater stability, compared to an unbiased parasite (Figs. 5A, 5B). In another case which includes demographic sex differences, male-biased parasitism results in greater stability at high fecundity, but is less stable at low fecundity (Figs. 5C, 5D). The dynamics of the corresponding uninfected populations are shown in Figures 1B and 1D. Finally, a male-biased system may be relatively less stable at all fecundities (results not shown).

**Figure 6:**
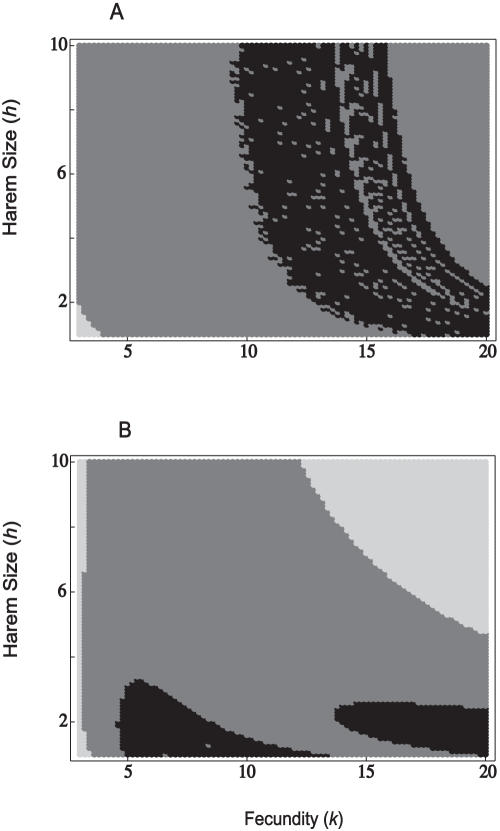
Stability plots, under the assumption of no host self-regulation. Regions shaded light grey correspond to a stable equilibrium, dark grey regions correspond to 2-point limit cycles, and regions shaded black correspond to higher-period cycles or chaotic dynamics; (A) β*_f_* = β*_m_* = 2.5; (B) β*_f_* = 1.25 and β*_m_* = 3.75. Other parameters are: α = 0.495, γ*_1_* = 0, γ_2_ = 0.005 and δ = 1.

Male-biased parasitism may therefore lead to either more stable or more complex dynamics; this may be dependent on the mating system and the case mortality. As a final example, we consider a population with no density-dependent self-regulation on its growth rate, as in the model of May [33]. In terms of our model, this is equivalent to μ*_f_* = μ*_m_* = 0. Figure 6 shows how the dynamical outcome is dependent on fecundity and mating system (harem size) by partitioning the (*k*, *h*) parameter space into regions where the dynamics reach a point equilibrium, a two-point cycle, or more complex dynamics (higher point cycles or chaos). We assume an extremely high mortality of infected hosts (χ = 0.99), but if hosts do recover then they cannot be re-infected (γ_1_ = 0). For an unbiased parasite, the dynamics attain a point equilibrium only in fully monogamous populations, and then only at very low fecundity. At higher harem sizes we observe either two-point cycles or, at certain high fecundities, more complex dynamics (Fig. 6A). Under male-biased parasitism, at the minimum fecundity the dynamics always reach a stable point, regardless of harem size. At higher fecundities we observe either two-point cycles or (at moderately low harem sizes) more complex dynamics. A high degree of polygyny together with high fecundity results in a point equilibrium (Fig. 6B).

## DISCUSSION

Sexual populations may exhibit complex dynamics independently of the effects of disease [26]. Parasitic infection may also result in complicated dynamical behaviour [33]. In this study we have concentrated on the differences between male-biased and unbiased parasitism, in terms of the population's dynamics. At low case mortality, we found there is unlikely to be a significant difference due to male-biased parasitism. This is likely to be because the majority of infected hosts recover, and the difference in male and female densities between cohorts is therefore marginal. At higher case mortalities, male-biased parasitism may result in qualitatively different dynamics from those in the equivalent unbiased model. The outcome has been shown to depend on a variety of factors (mating system, case mortality, demographic sex differences).

The potential for an Allee effect [36], [37] is implicit in our choice of birth function (equation 9). This is essentially a cost of rarity for sexual populations, due to scarcity of breeding partners [38], [39]. At low density, sexual populations may therefore be expected to have reduced rates of reproduction. Population growth rates may also be reduced at low density due to associated factors, such as social dysfunction and inbreeding depression [40]. In the absence of infection, an uneven sex ratio may destabilize the dynamics: period-doubling bifurcations are a common feature of the disease-free system [26]. In the host-microparasite model presented here, an Allee effect is theoretically possible whenever male-biased parasitism results in an uneven sex ratio.

Unbiased parasitism may often stabilize a population's dynamics (Fig. 2). Where a proportion of the population dies from infection, this reduces both the male and female densities and therefore the number of births. As such, infectious disease may stabilize the population by reducing its overall growth rate. At high case mortalities, both male-biased and unbiased parasitism are compatible with limit cycles (Figs. 3–6). At extremely high case mortality, the population may exhibit high-point cycles or chaos (Fig. 6). Population cycles are a feature of continuous models where disease transmission includes a free-living infective stage [28], [35]. In particular, cycles are predicted when the increased mortality rate due to infection (virulence, α) is high. This agrees with our results, in that we found cycles at high case mortalities. Limit cycles are also predicted at high case mortality in the discrete asexual model [33], [34]. During a large epidemic, a highly virulent pathogen reduces the male and female densities to low levels. The following epidemic is therefore much smaller, with a greater density of hosts surviving to reproduce. This allows the population to recover and triggers another epidemic. In the long-run then, the population oscillates between high and low densities [33], [34].

There is considerable evidence for male-biased parasitism in wild populations of vertebrates [1], [2], [7]. This phenomenon may also extend to contemporary human populations, since men are more than twice as likely to die from parasitic and infectious diseases than are women [41]. However, the mechanisms underpinning these biases are not well understood. Parasite levels are determined by the interaction between *exposure* to parasite infective stages and *susceptibility* to infection following exposure. Increased male susceptibility to parasites is often attributed to the immunodepressive effects of the androgenic hormone, testosterone; the so-called immunocompetence handicap hypothesis [2], [8], [42]. However, evidence supporting a link between testosterone levels and parasite susceptibility remains equivocal [43], [44]. Increased male susceptibility to parasitism may also be due to trade-offs between the levels of limiting resources allocated to sexually selected traits (including large size) and those allocated to immune defence [7], [45]. Increased male exposure to parasites may be mediated by a variety of mechanisms. In mammals, male-biased parasitism is associated with sexual size dimorphism, with males being the larger and more heavily parasitized sex [7], [46]. Large body size may lead to increased parasite exposure by virtue of offering a larger target for the parasite infective stages to exploit [7]. Large males may also have greater exposure to parasites, due to their larger home ranges and increased activity levels [7; but see 41]. Regardless of the exact mechanisms generating male-biased parasitism, there is convincing evidence that males may also be responsible for the majority of disease transmission. Perkins et al. [3] investigated the role of key hosts in the yellow-necked mouse, *Apodemus flavicollis*. These are parasitized by the sheep tick, *Ixodes ricinus*, the vector of the zoonotic tick-borne encephalitis (TBE) [47]. Sexually mature males of high body mass were identified as a functional group responsible for driving most of the transmission. Removal of this group (which constituted 26% of the total population) was predicted to reduce transmission potential by 79%. In another study on *A. flavicollis*, Ferrari et al. [4] experimentally reduced the helminth community to either sex, of the dominant macroparasite nematode, *Heligmosomoides polygyrus*. Reducing the parasite intensity of males significantly reduced the intensity in females, estimated through faecal egg counts, although reducing the intensity in females had no significant effect on the intensity in males. Furthermore, 20% of the most infected individuals (62% of which were males) were found to be responsible for 73% of the total eggs expelled. These two studies both roughly conform to the ‘20/80 Rule’, by which 20% of the individuals account for 80% of the parasite's transmission potential [48]. Male-biased parasitism may often be responsible for disease persistence, by maintaining the basic reproductive ratio (*R*_0_) of the pathogen above unity [3]. In our study, male-biased parasitism was modelled as increased relative susceptibility. Once infected though, males may also cause the majority of the transmissions to females, as it seems likely that the most heavily parasitized group will be most responsible for infecting others [6]. That being said, males may be intrinsically more infectious, due for example to their increased activity and/or host range. Investigating the effect of a high male susceptibility and infectiousness may form the basis of future work.

Our model makes a number of biological assumptions, which may limit its applicability. For example, we assume that males have greater susceptibility to infection but do not exhibit any other differential effects. However, increased susceptibility to infection may often be accompanied by higher virulence, as a result of pathogen replication in hosts [29], [49]–[50]. Where males have greater susceptibility, this may be due to weaker immune function [9], [51]–[53], which may also sometimes correlate with a higher virulence. Reductions in mating rate and fecundity due to parasitic disease may also occur. Sterilization effects are associated with sexually transmitted diseases in particular [54], and may often affect the sexes differentially. Our choice of birth function also implicitly assumes random mating among healthy and parasitized individuals. However, females may show preferential mating with regard to unparasitized males [55], [56]. As outlined earlier, our model assumes non-overlapping generations such that all individuals are either susceptible or recovered (or deceased) before reproduction occurs, but it would be interesting to investigate the implications of overlapping generations. However, this would necessarily complicate the analysis and our aim here has been to identify a difference rather than to quantify it for a particular system.

The aim of this study was to examine the effect on dynamical stability of male-biased parasitism. At low case mortality there appears to be little difference compared to an unbiased parasite. At higher case mortality, male-biased and unbiased parasitism may exhibit differential effects on the host population's dynamics. The outcome is influenced by the type of mating system, and demographic sex differences can also have an effect. Our central finding, that male-biased parasitism may result in different population dynamics compared to an unbiased parasite, may hopefully provide a useful basis for further research. Male-biased parasitism is increasingly being identified in ecological systems, and our model can be adapted to fit these. This may lead to some interesting theoretical predictions, which could be measured against real systems.
